# Supplementary material for: An entropic barriers diffusion theory of decision-making in multiple alternative tasks
Source: PLoS Comput Biol. 2018 Mar 2;14(3):e1005961. doi: 10.1371/journal.pcbi.1005961 (PMC5851639; doi:10.1371/journal.pcbi.1005961)
Supplement: S1 File — Analytic solutions of First Passage Time distributions. Individual player and automata RT distributions. (PDF) [file pcbi.1005961.s001.pdf]

An entropic barriers diffusion theory of decision-making in multiple alternative tasks (Supplemental material)

Diego Fernandez Slezak[1], Mariano Sigman[2], and Guillermo A. Cecchi[3]

[1]Laboratorio de Inteligencia Artificial Aplicada, Departamento de Computación, FCEyN, UBA, Pabellón 1, Ciudad Universitaria, (C1428EGA) Buenos Aires Argentina, CONICET
[2]Universidad Torcuato Di Tella, Alte. Juan Saenz Valiente 1010, Buenos Aires C1428BIJ, Argentina, CONICET
[3]Computational Biology Center, T.J. Watson Research Center, IBM, P.O. Box 218, Yorktown Heights, N.Y. 10598, USA

1 Analytic solutions of First Passage Time distributions

There is a vast body of literature on analytic solutions for first-passage times in simple geometries. An excellent source is the book by Redner [S. Redner, A guide to first-passage processes, Cambridge University Press, New York (2001)] . For a semi-finite interval with boundary at $x = 0$ and seed at x_0 the FPT distribution is:

$$W(t) = \frac{x_0}{\sqrt{4\pi D t^3}} e^{-x_0^2/4Dt} \tag{1}$$

whereas for a finite interval of size L and seed at $x = L$

$$W(t) = \frac{2\pi D}{L^2} \sum_{n=0}^{\infty} (n + \frac{1}{2}) \sin\left(\pi(n + \frac{1}{2})\frac{x_0}{L}\right) e^{-\pi^2(n+\frac{1}{2})^2 Dt/L^2} \tag{2}$$

It can be readily seen that the distribution for the semi-finite interval is dominated by a power-law tail $t^{3/2}$, which preserves the functional form for higher dimensions, leading to $t^{d/2+1}$. The finite interval, on the other hand, is dominated by an exponential decay. A more interesting geometry for the purpose of our model is that of a comb with infinite side-branches, in which the seed is located at one extreme of the comb's backbone, the sink at the other extreme

at a finite distance L, and a number of infinite branches are located at regular intervals [1]. An approximate solution for this case is

$$W(t) \sim \frac{1}{t^{1/2}} e^{-t^{1/2}/L^2} \tag{3}$$

We show in Fig. 1 a best fit of the comb model to the data. As it can be seen, the comb model provides a reasonable fit, but it is not as accurate as the obstacle model. In particular, the approximate solution is only valid for the tail, as it cannot fit the distribution near the origin.

2 Individual player distributions

To verify that super-exponential distributions are an intrinsic behavior of multiple-alternative decision-making – and not produced by the sum of exponential distributions corresponding to many classic decision-making models – we fitted individual players. We selected players with more that 20.000 games each, concluding in 16 players. For each player, we performed the model fitting considering entropic barriers. As an example, in figure 2 we show the result of fitting 9 players individually at 50% of the game. Blue line shows the human data, and green line shows the best fit curve obtained from the model. All players showed an almost perfect fit (KS test, $p < 10^{-8}$ in all cases).

Then, we splitted each player data into 80%-20% training-test datasets, randomly chosen. Using the 80% subset we fitted the models and tested the fit by calculating de JSD in the 20% test dataset. We repeated this procedure 1000 times and obtained the average JSD value for each player fitting both models (see Fig. 3). We observe that obstacles model show better performance than DDM for all players in the cross-validation scheme.

3 Automata RT distributions

We presented the RT statistics for human players and showed that decision RTs are shorter during the first and last stages of the game, with each RT distribution characterized by a super-exponential tail. A similar statistics analysis for games played by computer agents shows marked differences (Fig. 4). Most computers showed time distributions are compressed towards shorter RT's, showing a bimodal behavior, with a peak close to zero and a second peak in the order of seconds depending on the observed instant of game. This fact is in marked contrast with the uni-modal characteristic of the human players RT's.

References

[1] Sidney Redner. *A guide to first-passage processes.* Cambridge University Press, 2001.

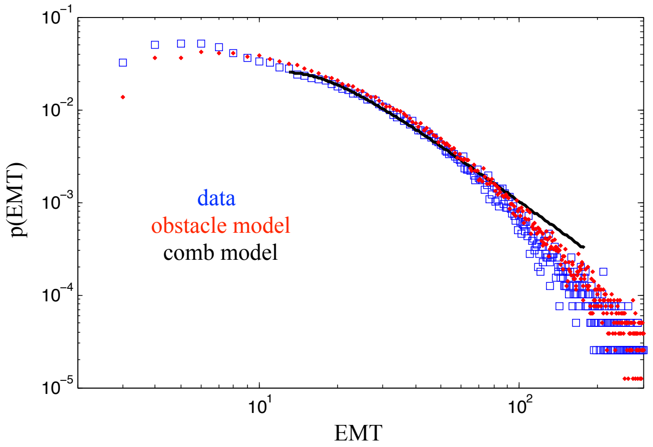

Figure 1: **Comparison with diffusion on a comb.** The figure shows the best fit to human RT (blue) of our diffusion with obstacles model (red), and an infinite comb model (black).

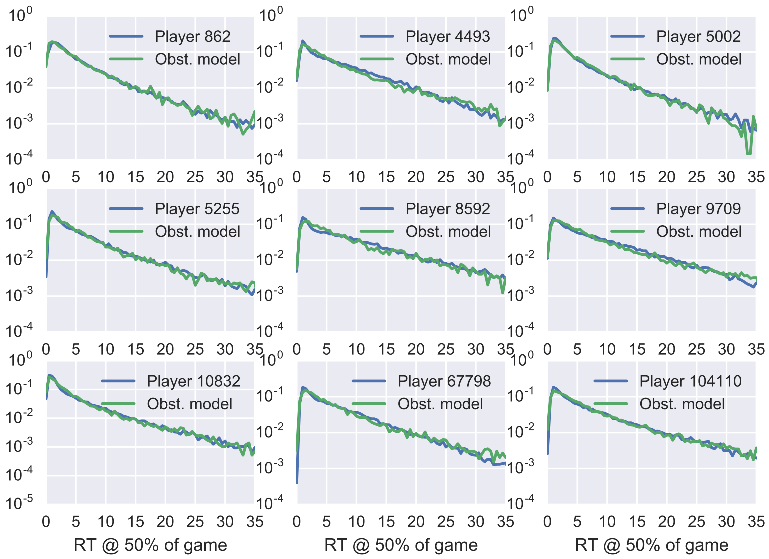

Figure 2: **RT distribution and model fit of individual players.** The panels show the RT distribution of 9 players (with more than 20000 games) at 50% of the game, with their corresponding best fit. Black line shows the human data and red line shows the best fit.

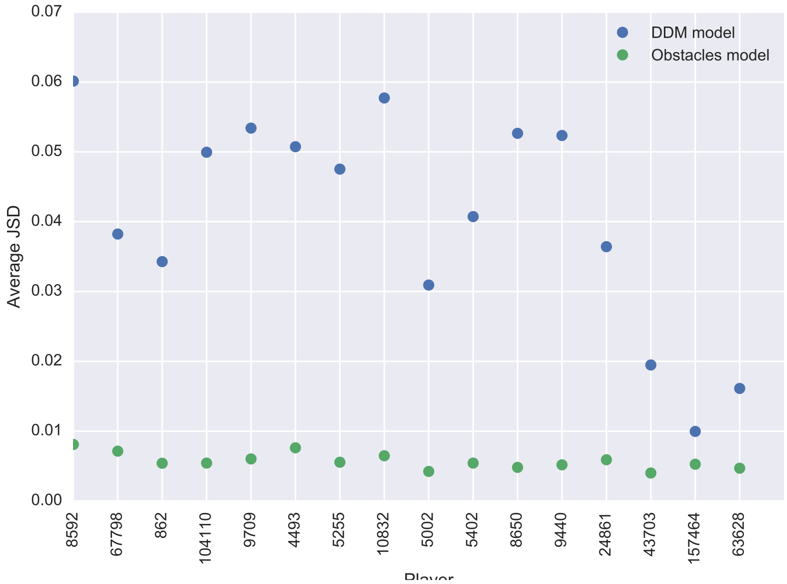

Figure 3: Average JSD values for most-active players in a Cross-validation scheme.

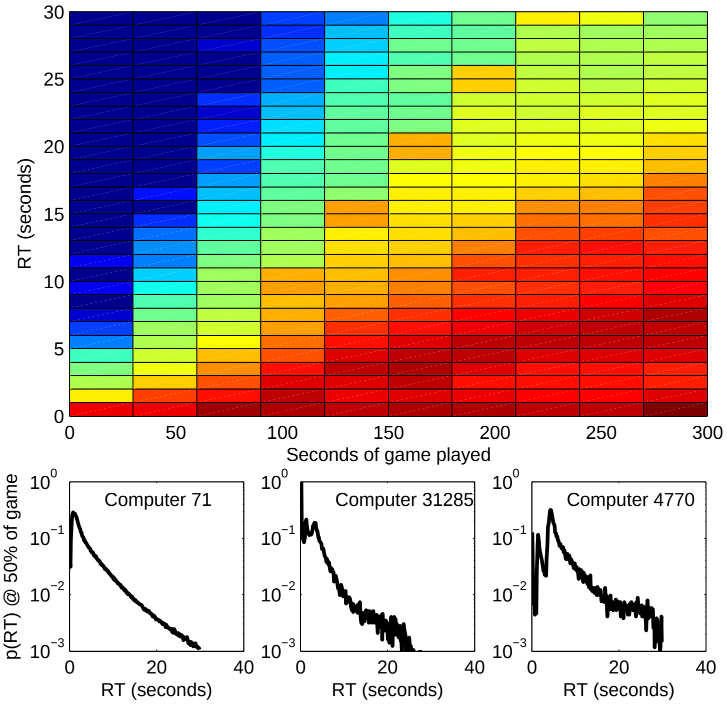

Figure 4: **Response Time statistics for automaton players.** Top panel: Histogram of RT as a function of the seconds of game played for computer players. Lower panels: panels show the RT distribution of 3 computer players (with more than 20000 games) at 50% of the game.
